# Optically-Monitored Nanopore Fabrication Using a Focused Laser Beam

**DOI:** 10.1038/s41598-018-28136-z

**Published:** 2018-06-27

**Authors:** Tal Gilboa, Adam Zrehen, Arik Girsault, Amit Meller

**Affiliations:** Department of Biomedical Engineering, The Technion – Israel Institute of Technology, Haifa, 32000 Israel

## Abstract

Solid-state nanopores (ssNPs) are extremely versatile single-molecule sensors and their potential have been established in numerous biomedical applications. However, the fabrication of ssNPs remains the main bottleneck to their widespread use. Herein, we introduce a rapid and localizable ssNPs fabrication method based on feedback-controlled optical etching. We show that a focused blue laser beam irreversibly etches silicon nitride (SiN_x_) membranes in solution. Furthermore, photoluminescence (PL) emitted from the SiN_x_ is used to monitor the etching process in real-time, hence permitting rate adjustment. Transmission electron microscopy (TEM) images of the etched area reveal an inverted Gaussian thickness profile, corresponding to the intensity point spread function of the laser beam. Continued laser exposure leads to the opening of a nanopore, which can be controlled to reproducibly fabricate nanopores of different sizes. The optically-formed ssNPs exhibit electrical noise on par with TEM-drilled pores, and translocate DNA and proteins readily. Notably, due to the localized thinning, the laser-drilled ssNPs exhibit highly suppressed background PL and improved spatial resolution. Given the total control over the nanopore position, this easily implemented method is ideally suited for electro-optical sensing and opens up the possibility of fabricating large nanopore arrays *in situ*.

## Introduction

The development of synthetic solid-state nanopores (ssNPs) as a substitute for biological channels remains a major focus in nanotechnology given their greater flexibility in terms of size, shape, surface properties, and cross-device compatibility^1–3^. While traditionally the principal mode of single-molecule detection was based on ionic resistive pulsing measurements, a rapidly growing trend in the nanopore community has been towards “electro-optical” sensing^4^. The simultaneous measurement of the electrical (ionic current) and fluorescent signals (photon emission) extends the scope of biomolecular targets for nanopores and opens up new applications since both multiple fluorophore colors and varying photon intensities can be acquired to obtain specific information on the molecule of interest. Specifically, by selective fluorescent labelling of the analyte of interest, researchers have shown that ssNPs can be applied to DNA sequencing, DNA barcoding, epigenetic modification analysis, and DNA methylation quantification^5–11^. Although superior to strictly electrical sensing with respect to the amount of encodable information, electro-optical sensing brings its own set of fabrication challenges. Nanopores must be prepared in a way such that their position can be readily identified *in situ*^12,13^. Furthermore, the peripheral structure heavily impacts the background noise and fluorescent signal of a translocating molecule^8,14,15^.

In the first decade of nanopore sensing, the controlled focusing of an ion or electron beam, as by transmission electron microscopy (TEM), was the only practical method for forming ssNPs with nanometric dimensions^16–21^. As these methods utilized high vacuum during pore drilling, it followed that they were inherently slow, expensive, and importantly, produced un-hydrated surfaces that must be further treated to permit water passage and subsequent resistive pulse sensing. More recently, controlled dielectric breakdown (CBD) emerged as a powerful, low-cost alternative to TEM because it could create nanopores in freestanding silicon nitride (SiN_x_) directly in solution and could be almost fully automated^22^. CBD, which uses an applied voltage to induce randomly accumulating material defects, is nonetheless comparatively less flexible and efficient at localizing nanopore formation^23^. Recent attempts to do so relied on the principle that nanopores preferentially form at the hotspot of an infrared (IR) laser^24^ or at the thinnest membrane cross-section^25,26^. Thus, in the latter case, milling^25^ or lithographic^26^ steps were implemented upstream of CBD as a preparatory step to direct nanopore formation. Using an IR laser, on the other hand, was complicated by the need to simultaneously control the applied voltage and laser power, as the IR laser only enhanced the local DC field necessary for dielectric breakdown and did not independently form nanopores.

Herein, we introduce a fast and highly reproducible method for fabricating nanopores based on feedback-controlled etching of freestanding SiN_x_ using a milliwatt-intensity blue laser. First we show that a focused 488 nm laser can locally etch freestanding SiN_x_ directly in solution, and that the emitted photoluminescence directly correlates with the local membrane thickness. We find that the etch rate closely follows the intensity point spread function (PSF) of the laser beam, resulting in an approximately inverted Gaussian thickness profile, as confirmed by TEM analysis. Next we demonstrate that with further laser exposure, the membrane thins to the point of nanopore formation, and that monitoring the current in parallel enables us to precisely control the nanopore size. This unique fabrication strategy offers several key advantages: First, pores can be rapidly formed at any arbitrarily-chosen location or multiple locations along the SiN_x_ membrane. Second, the drilling process is well-controlled via the laser intensity; hence, it can be utilized to tune the pore diameter. Finally, due to the localized thinning, the ssNPs display a larger signal amplitude and a highly suppressed background PL, optimal for electro-optical measurements.

## Results and Discussion

### Laser-Etching of Freestanding SiN_x_

**Figure 1:**
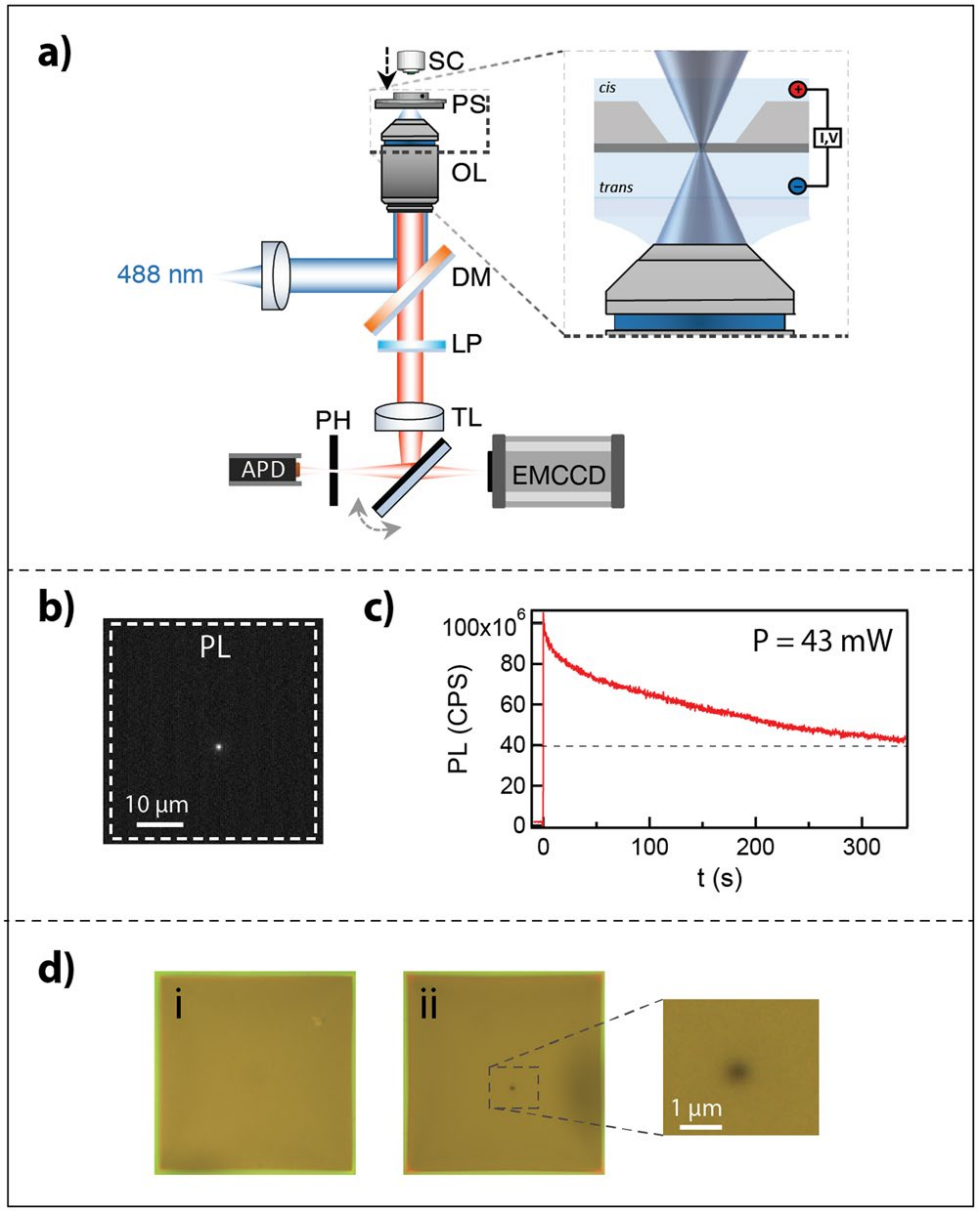
Laser thinning of freestanding SiN_x_. (**a**) Schematic of the confocal setup. SC- SiN_x_ chip; PS-piezo stage OL- objective lens; DM- dichroic mirror; LP- long pass filter; TL- tube lens PH- pinhole. The emission pathway is switchable between the APD and EMCCD. (**b**) Focusing of a ~45 mW 488 nm laser on the membrane results in photoluminescence emission, which is recorded by the APD in the >550 nm range. (**c**) Photoluminescence emission during laser-exposure, measured in counts per second. The laser is activated at t = 0 seconds. (**d**) Images of the 42 × 42 μm^2^ membrane under white-light illumination before etching (i). After 300 seconds of laser exposure, a thin region is visible as a contrasted spot (ii).

We first developed a procedure for etching freestanding SiN_x_ with a continuous-wave blue (488 nm) solid-state laser. It begins by assembling a Si-supported SiN_x_ membrane (typically 40–45 nm thick) in an optically accessible flow cell, which is then mounted on top of a high NA microscope objective in a homebuilt confocal setup (Fig. 1a). The setup is equipped with an EMCCD for widefield viewing and an avalanche photodiode (APD) detector for high temporal resolution sensing of the photoluminescence (PL) intensity. For alignment, we set the blue laser at low intensity (40 µW) to prevent unintentional etching and bring the membrane into focus of the laser spot. Once aligned, the laser intensity is increased to full power (~45 mW) for the etching step.

Notably, we observed that under high laser intensity, a bright PL emission was visible by our EMCCD camera (Fig. 1b). The measured PL intensity exhibited a decay over time before reaching a near plateau level after roughly 300 s (Fig. 1c). We confirmed that the decay in PL is not due to mechanical drift and is in fact irreversible: momentarily switching off the laser beam and then switching it on again showed that the PL level retuned to the same level at which the laser was switched off (and not to the initial level). Furthermore, widefield optical inspection of the membrane revealed a darkened spot at the point where the material was illuminated by the laser (Fig. 1d). The material darkened proportionally to the PL reduction, and this spot could not be revived by solvent or acid cleaning.

**Figure 2:**
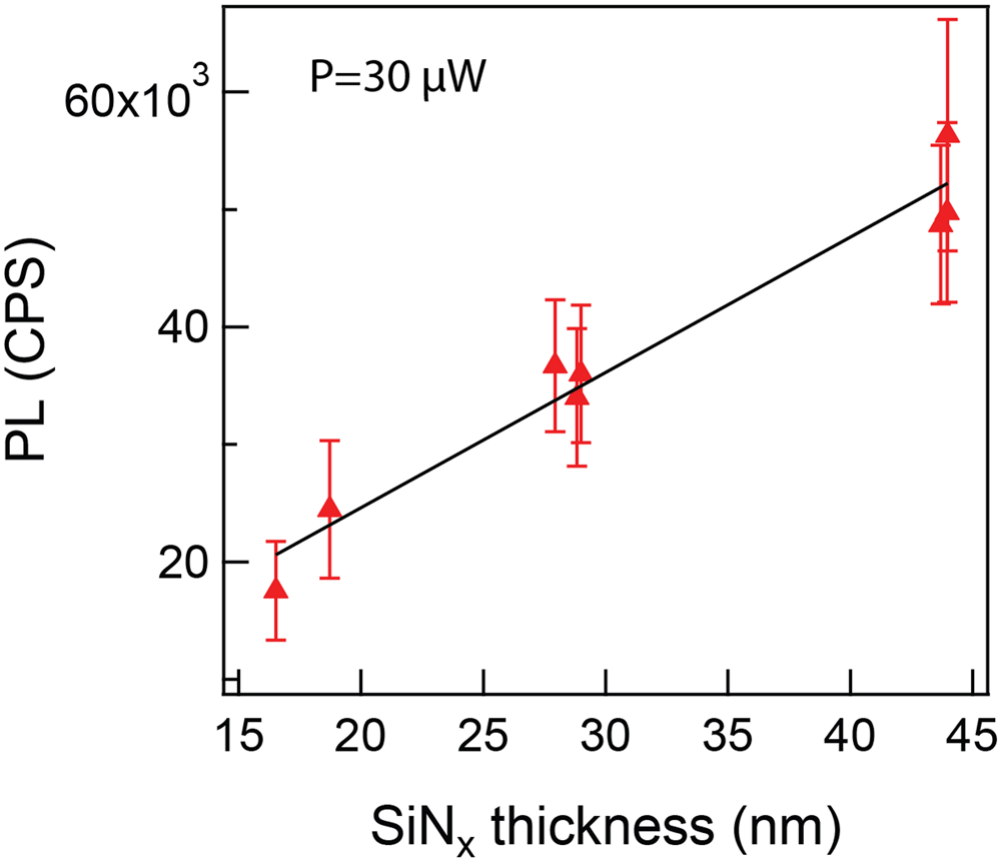
Photoluminescence (PL) intensity calibration as a function of SiN_x_ thickness. The PL in counts per second (CPS) was measured by the APD during laser-exposure (488 nm, 30 µW) for 6 chips of different membrane thickness. Prior to the PL measurements, the membrane thickness was measured by ellipsometry. Thicker membranes result in higher PL.

As contrast under white-light illumination typically indicates a difference in material thickness^27^, to further characterize this phenomenon we fabricated a series of freestanding SiN_x_ membranes from the same stock material, using reactive ion etching (RIE) to obtain different final thicknesses. Accurate thickness measurements were made by ellipsometry after performing a careful calibration using a factory-supplied model specimen. The chips were then mounted in our optical setup and the PL level was determined under otherwise identical conditions. Our results, summarized in Fig. 2, show a linear relationship between PL and the SiN_x_ membrane thickness. We note that, as expected, the measured PL intensity varies sharply with the distance between the objective lens and the membrane, and reaches a maximum value when the laser spot is centered in the *z* direction on the membrane^11^. Hence the measurements shown in Fig. 2 involved careful maximization of each PL read in the *z* direction. Measurements were performed using an attenuated laser (30 µW) to avoid etching of the membrane and remain constant over time. The error bars reflect the standard deviation in the PL intensity over 1 minute of measurement.

**Figure 3:**
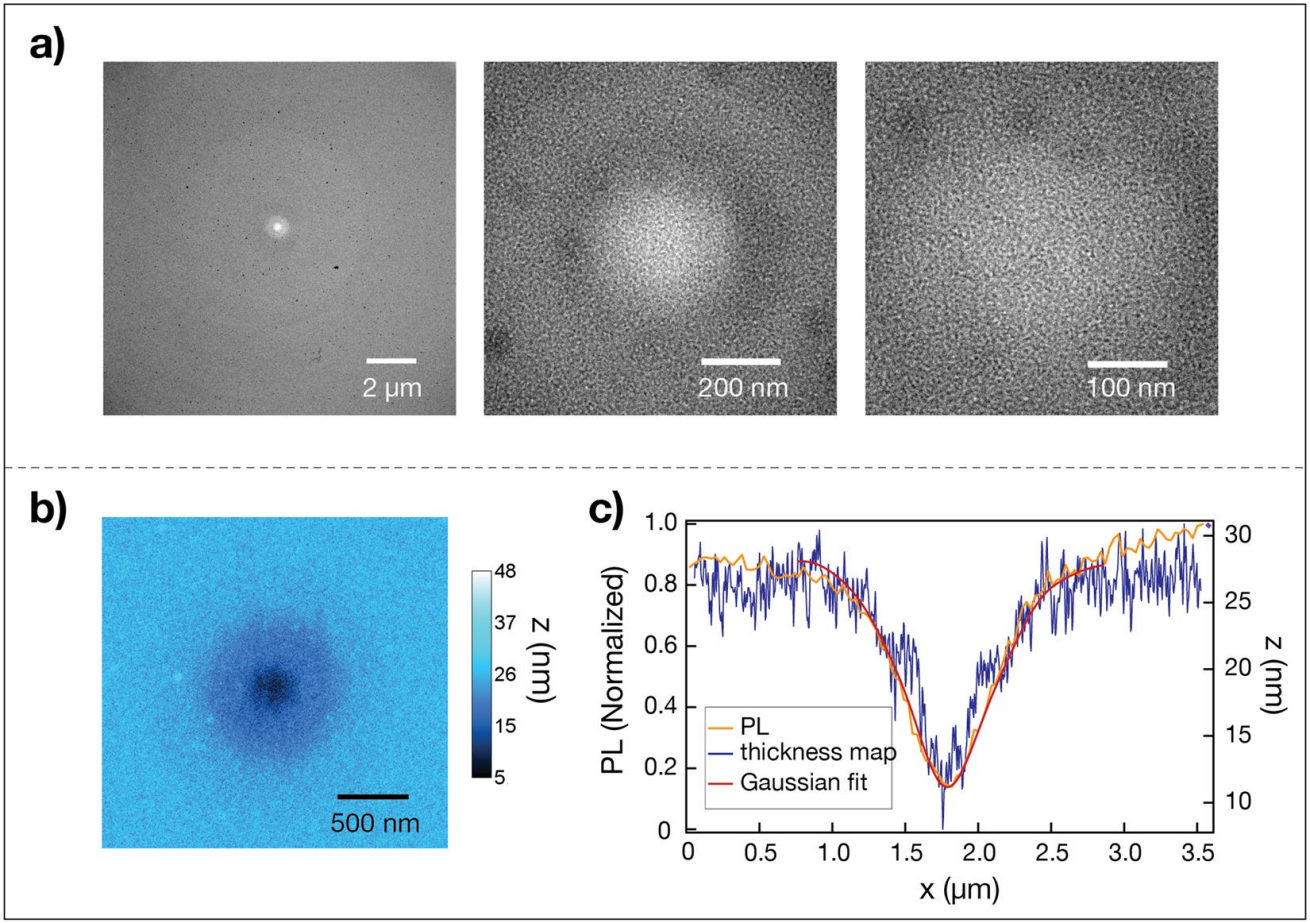
Thickness characterization of a laser-etched spot. (**a**) TEM images at 195×, 39000× and 75000× (left to right). The lighter region corresponds with higher transmittance and thus thinner material. (**b**) TEM thickness map of an etched spot in nanometers. (**c**) Blue curve- TEM thickness map. Purple curve- normalized photoluminescence (PL) scanned in the *x* direction with a 30 nm step size. Red curve- simulated normalized PL based on a convolution of a diffraction-limited Gaussian, representing the laser beam, with the TEM thickness map.

We next imaged the samples by Transmission Electron Microscopy (TEM) to determine whether the darkened membrane spot caused by the laser was in fact due to material removal and not a type of laser-induced chemical reaction or adsorption process. Indeed, the TEM images reveal that the material had thinned at the position of the laser focus (Fig. 3a, Supplementary Fig. S1). Moreover, the TEM images show that the material thins non-uniformly: the thickness profile closely follows the intensity point-spread function (PSF) of the laser beam used to induce thinning, where etching occurs fastest at the center (Fig. 3b). See Methods for a description on making the TEM thickness map.

After establishing that the laser etches SiN_x_, we formulated a relationship between measured PL and etch depth which is consistent with the TEM analysis. After two minutes of laser exposure, we lowered the laser intensity to prevent etching and scanned in the *x* direction with a 30 nm step size while measuring PL intensity. The generated 1D PL profile and TEM thickness map were both normalized and overlaid on the same graph (Fig. 3c, orange and blue curves, respectively). As can be seen, the PL curve matches the true SiN_x_ thickness, deviating slightly because of practical limitations of the optical setup. We simulated a PL curve based on a convolution of a Gaussian PSF, representing the laser beam, with the TEM thickness map (Fig. 3c, red curve). Overlaying the modelled data on the same graph shows a tight fit with the PL measurement, with a PSF full width at half maximum (FWHM) of 325 ± 15 nm. This compares favorably with the diffraction-limited FWHM of 330 ± 20 nm for PL emission collected by an objective lens with a numerical aperture (NA) of 1.15. Therefore, we can reliably use the PL measurement to infer the membrane thickness.

We found that the etch rate is significantly reduced at low laser intensity and is practically undetectable for 488 nm laser intensities <1 mW over the course of our measurements. The etch rate for the 488 nm laser at an intensity of ~45 mW was found to be up to 25 nm/minute. Interestingly, red laser (645 nm) induced no appreciable membrane thinning over a similar timescale, while green laser (532 nm) focused on the membrane at the same power as the 488 nm laser, resulted in roughly an order of magnitude less thinning, indicating that the etching mechanism is dependent not only on the laser intensity but also on its wavelength (Supplementary Fig. S2). This is consistent with a previous study which did not report any membrane thinning despite using a comparable laser power (~45 mW, 785 nm)^24^. Interestingly, we found that etching also proceeds in ultrapure water (18.2 MΩ × cm) and not just in KCl buffer. Although SiN_x_ etching in water has been reported before in the literature^28^, it required the use of sub- or super-critical water with temperatures in the 200 °C range and a pressure of 10 MPa. Our finding that the 532 nm laser produced much less SiN_x_ thinning than the 488 nm laser at the same power suggests that the etch process is likely not temperature-activated but rather follows some form of wavelength-dependent photochemical etching. It is known that differences in laser-etching rates is a consequence of differences in spatial-electron hole pair density, which is a function of their respective absorption coefficient for a particular material^29–31^.

### Nanopore Fabrication and Validation

**Figure 4:**
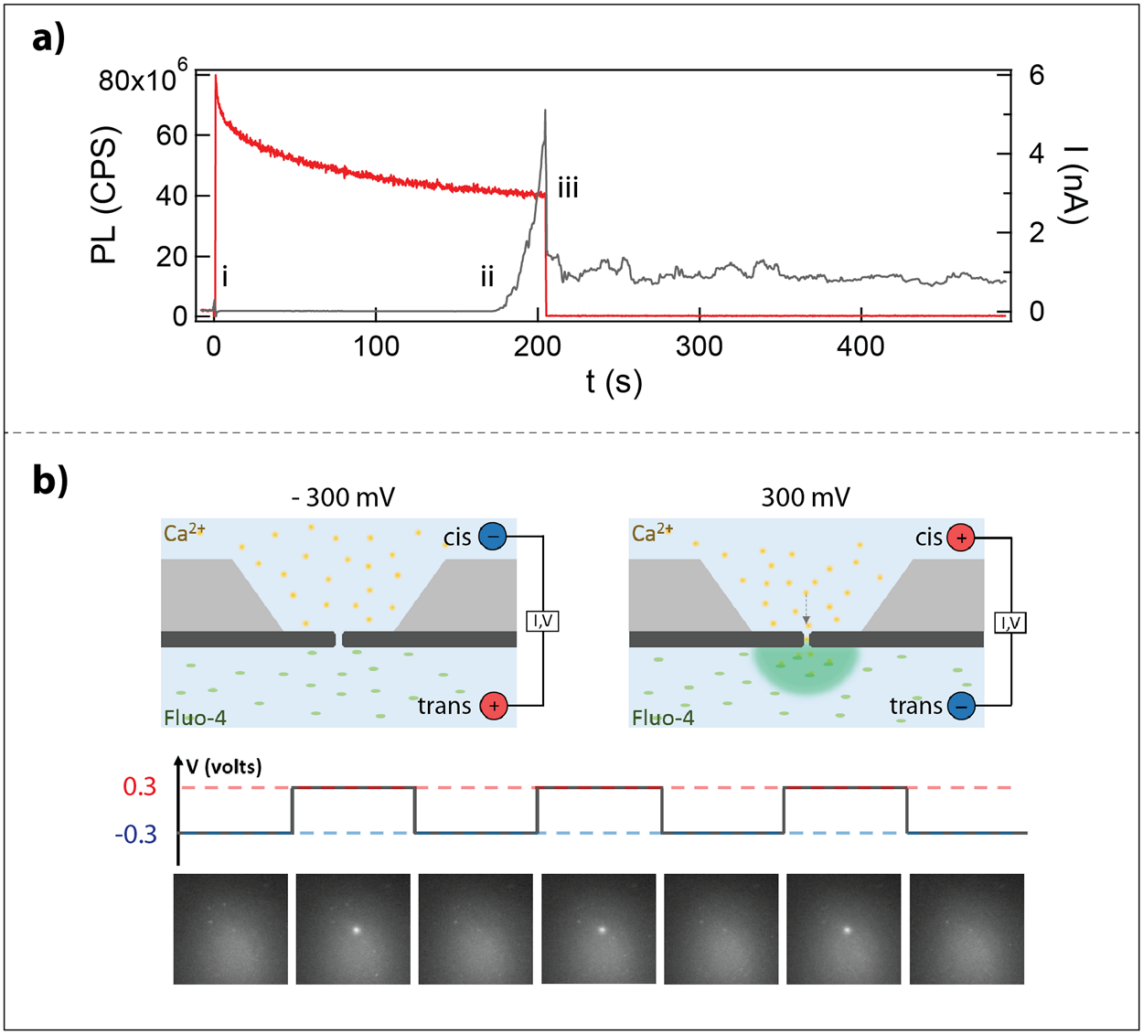
Nanopore fabrication by laser-etching. (**a**) Measured photoluminescence (PL) and ionic current during laser-exposure (red and grey curves, respectively). The PL sharply increases when the laser is activated (i). Pore formation is signaled by an increase in current (ii). Following ~20 s of pore growth under continued laser-exposure, the laser is deactivated, and the PL returns to zero (iii). Turning off the laser causes a conductivity decrease, resulting in a coincident drop in current which stabilizes over time. (**b**) Principle of calcium (Ca^2+^) activators used for verifying the creation of a nanopore (top panels). The entire membrane is illuminated by a 488 nm laser. At −300 mV, Ca^2+^ is driven away from the pore. At +300 mV, Ca^2+^ is driven through the pore where it binds to Fluo-4 resulting in detectable fluorescence at >510 nm. The bottom panels show calcium activators applied to laser-drilled pores. The bias is repeatedly switched between positive and negative to validate the presence of a nanopore.

Based on our observation that a ~45 milliwatt-intensity blue laser etches SiN_x_, we attempted to fabricate nanopores by progressively thinning the membrane until the point of nanopore formation. For this, we monitored the ionic current across the membrane, applying a 300 mV transmembrane potential via *cis/trans* –immersed AgCl electrodes connected to an Axon 200B amplifier. We simultaneously measure the PL as a way to track the fabrication progress. An example experiment with concurrent ionic and PL feedback is given in Fig. 4a. In this example, we observed an increase in ionic current after roughly 145 seconds, which we attribute to the formation of an ionic passageway through the membrane. The current continues to rise until the laser is deactivated, which we associate with nanopore growth. Notably, upon pore formation the ~45 mW laser also causes an increase in electrolyte conductivity^32,33^, hence, turning the laser off causes the current to drop. The open pore conductance then stabilizes over the next few minutes, usually deviating at most 2 nS from its initial value. The final conductance level increases with the time that the laser is kept on after the initial formation of the pore.

**Figure 5:**
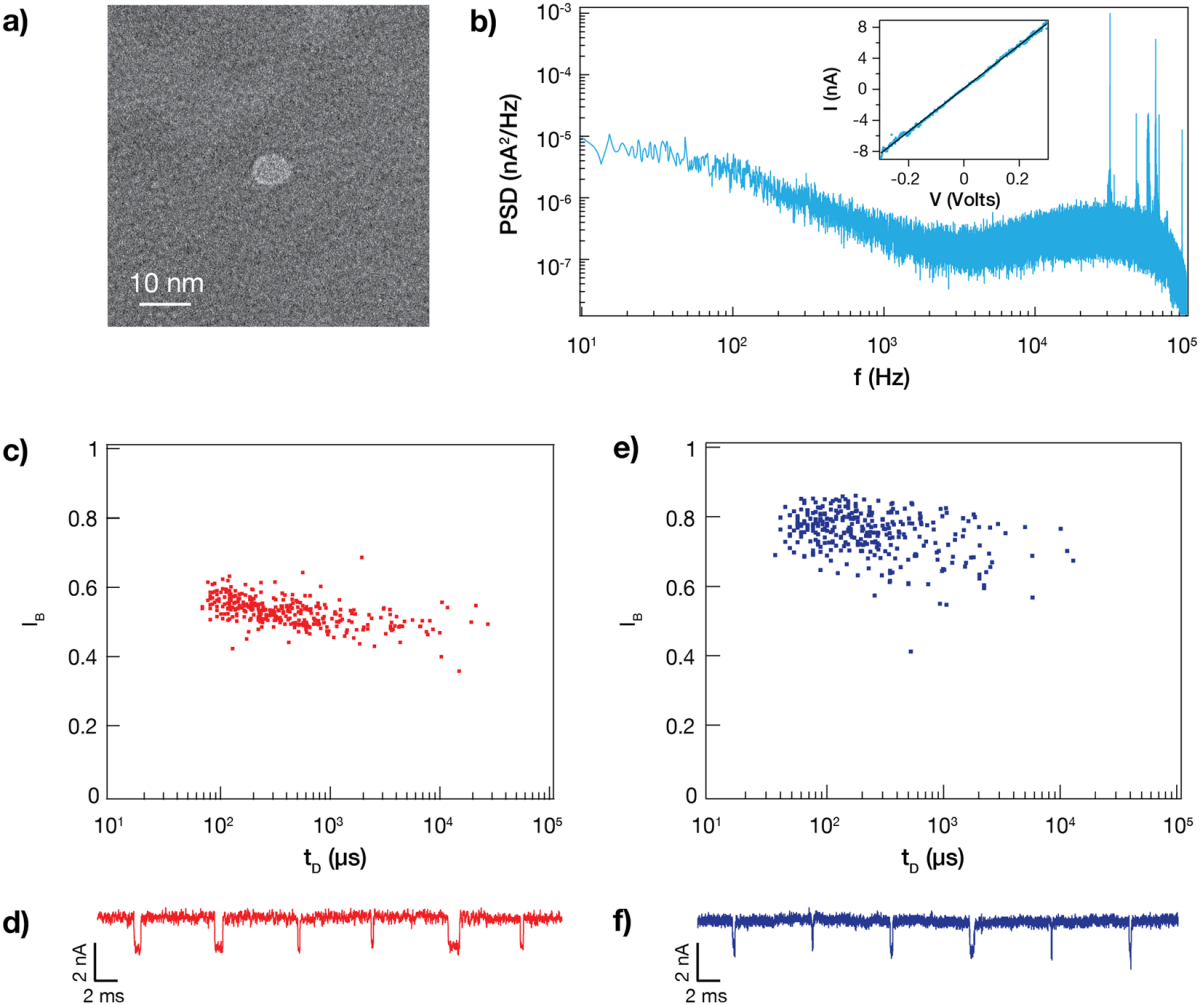
Noise and functionality of a laser-etched nanopore. (**a**) TEM image showing a nanopore with a diameter of 6.5 nm. Compared to the peripheral membrane, the nanopore is very bright, owing to an unobstructed electron beam path. (**b**) Power spectral density (PSD) plot of a nanopore for an applied bias of 300 mV. The inlet shows the corresponding current-voltage (IV) curve for this nanopore, with a linear fitting (R^2^ > 0.99). (**c**) Scatter plot of dsDNA translocation events. The *trans* chamber was biased to 300 mV to drive translocation of 300 pM 5054 bp dsDNA from *cis* to *trans*. The size of the pore is 3.1 ± 0.3 nm based on the current blockage level/molecular ruler model. (**d**) A concatenated ionic current trace showing sample dsDNA translocation events. (**e**) Scatter plot of di-ubiquitin (K63-linked di-Ub) translocation events at pH 7. The *trans* chamber was biased to 300 mV to drive translocation of 0.007 μg/µl di-Ub from *cis* to *trans*. (**f**) A concatenated ionic current trace showing sample di-Ub translocation events.

We first validated that a thoroughfare path was truly made in the membrane and that the measured current was not caused by surface charging or some other effect. To do so, we loaded the *cis* side chamber with calcium (Ca^2+^) and the *trans* chamber with Fluo-4, and illuminated the entire membrane at 488 nm while monitoring it with a CCD (Fig. 4b, upper panel)^26^. For there to be a path through the membrane, the fluorescence signal should sharply increase when the applied *cis/trans* bias is positive, as the Ca^2+^ would be driven through and activate Fluo-4. Indeed, as shown in Fig. 4b (lower panel), we observed a fluorescent signal at the exact position where the material was etched. We next sought to corroborate our calcium-imaging data with TEM data. After a laser drilling experiment, we allowed the OPC to stabilize for over 15 minutes. We then immersed the SiN_x_ chip in ultrapure water to remove salt residue. Figure 5a gives an example TEM image of a 6.5 nm nanopore formed in under 5 minutes, which was a typical fabrication time in a 40–45 nm thick membrane based on >30 trials (100% yield).

As we show, by choosing a current threshold for laser shutoff, we are able to reproducibly fabricate both small (1 nm) and large (over 10 nm) nanopores according to the sensing requirement (Supplementary Fig. S3, Table S1). Given that our nanopore fabrication strategy is markedly different than existing techniques such as CBD or TEM-drilling, we cannot expect that the standard conductance model^34,35^ for pore size determination applies; in particular, this model assumes an effective nanopore height equivalent to or one third of the membrane thickness, depending on the method employed^27,36,37^. Instead, we can reliably estimate the nanopore diameter according to the translocation blockage level using a molecular ruler of known dimensions, such as dsDNA (2.2 ± 0.1 nm), and the following equations^38^:

1 $${i}_{O}=\frac{V}{\frac{4l}{\pi {d}^{2}}+\frac{1}{d}}\sigma $$

2 $${I}_{B}\equiv \frac{{i}_{B}}{{i}_{O}}=(1-\frac{{a}^{2}}{{d}^{2}})$$

where ${i}_{O}$ and ${i}_{B}$ are the open and blocked pore current levels, respectively, *l* is the local membrane thickness, *d* the pore diameter, $\sigma$ the solution conductivity and *a* is the analyte diameter. To demonstrate the extent by which the conductance model needs to be adjusted, we calculated the effective thickness for a pore with an OPC of 11 ± 0.7 nS and a diameter of 3.2 ± 0.3 nm. Remarkably, we get an effective thickness of 4–6 nm, which is up to 11 times smaller than the surrounding membrane and is consistent with our observation that the membrane gradually thins to the point of nanopore formation. Such ultrathin architectures are highly desirable due to their larger conductance and hence improved spatial resolution, and have therefore been the subject of much research^39^.

We next evaluated the noise characteristics of laser-etched nanopores. Figure 5b shows the power spectral density (PSD) plot of a nanopore for an applied bias of 300 mV after allowing the nanopore to stabilize in KCl buffer. Similar to TEM-drilled nanopores, two sources of noise dominate the PSD: high-frequency background noise associated with the chip capacitance, and low-frequency flicker noise with 1/f^α^ dependency^40,41^. At an applied voltage of 300 mV, these nanopores typically exhibit an *i*_*RMS*_ in the range of 100–200 pA. To assess ionic current rectification, which occurs due to a geometric or surface charge asymmetry along the axis of current flow^42^, we varied the potential from −300 to +300 mV with equimolar salt concentrations in the *cis/trans* chambers. The resulting IV curve is linear (R^2^ > 0.99), indicating minimal rectification and therefore a symmetric geometry (Fig. 5b, inset). This suggests that the laser-induced etch mechanism occurs on both sides of the membrane equally to produce a very thin hourglass-shaped nanopore.

Finally, we validated the functionality of laser-etched nanopores by performing extensive sets of DNA and proteins translocation experiments. First, we added 300 pM 5054 bp dsDNA, produced and purified in house, to the *cis* chamber filled with KCl buffer. Upon biasing the *trans* chamber at +300 mV, the initially stable open pore was interrupted by current blockage events of 1.4–2.2 nA or 0.42–0.62 of the open pore current (Fig. 5c,d). For a pore of this small size (3.2 ± 0.3 nm), we can expect a significant fraction of events to be collisions, as has been established by both theory and experiment^39,43^. Therefore, to determine whether there are any full translocations, we performed an additional two translocation experiments at 450 and 650 mV and compared the dwell times of the three experiments. As can be seen in Supplementary Fig. S4, there is an obvious decrease in average dwell time with increasing voltage, indicating that a distinct portion of the events are successful translocations and not collisions. In a subsequent experiment using another pore, the Gaussian fitting clearly delineates two populations corresponding to two event types: short and low blockage/shallow events representing translocations, and long and high blockage/deep events representing collisions (Supplementary Fig. S5). The short and shallow events, though fewer in number, appear at the expected ratio relative to the long and deep events assuming that the DNA polymer behaves the same as it does with TEM-drilled nanopores^39,44^. Nevertheless, both nanopores studied generated sufficient events to produce a statistically reliable result^44^.

We further challenged our nanopore fabrication method to the purpose of detecting one of the smallest protein molecules (K63-linked di-ubiquitin, ~17 kDa), which compared to DNA, poses exceptional spatial and temporal resolution requirements. As has been demonstrated with TEM-drilled pores, one way to reduce the protein translocation rate is to use a buffer pH close to the isoelectric point (pI) of the protein^45–47^. Therefore, for di-ubiquitin (di-Ub) with a pI of 6.7^48^, we adjusted the KCl buffer to an experimentally determined^47^ pH value of 7. Using a nanopore with an OPC of 7–7.2 nA, we observed shallow (0.2 of the OPC) and mainly short (40–200 μs) events upon the addition of di-Ub to the *cis* chamber (Fig. 5e,f, Supplementary Fig. S6), expected for this pH value. This set of experiments proves that these nanopores are suitable not just for DNA studies but also small and compact proteins such as di-Ub. Moreover, we note that many of the ssNPs used for translocation experiments were over 10 days old—kept dry in air and made hydrophilic prior to the experiment—attesting to the high stability of laser-etched nanopores.

## Conclusions

**Figure 6:**
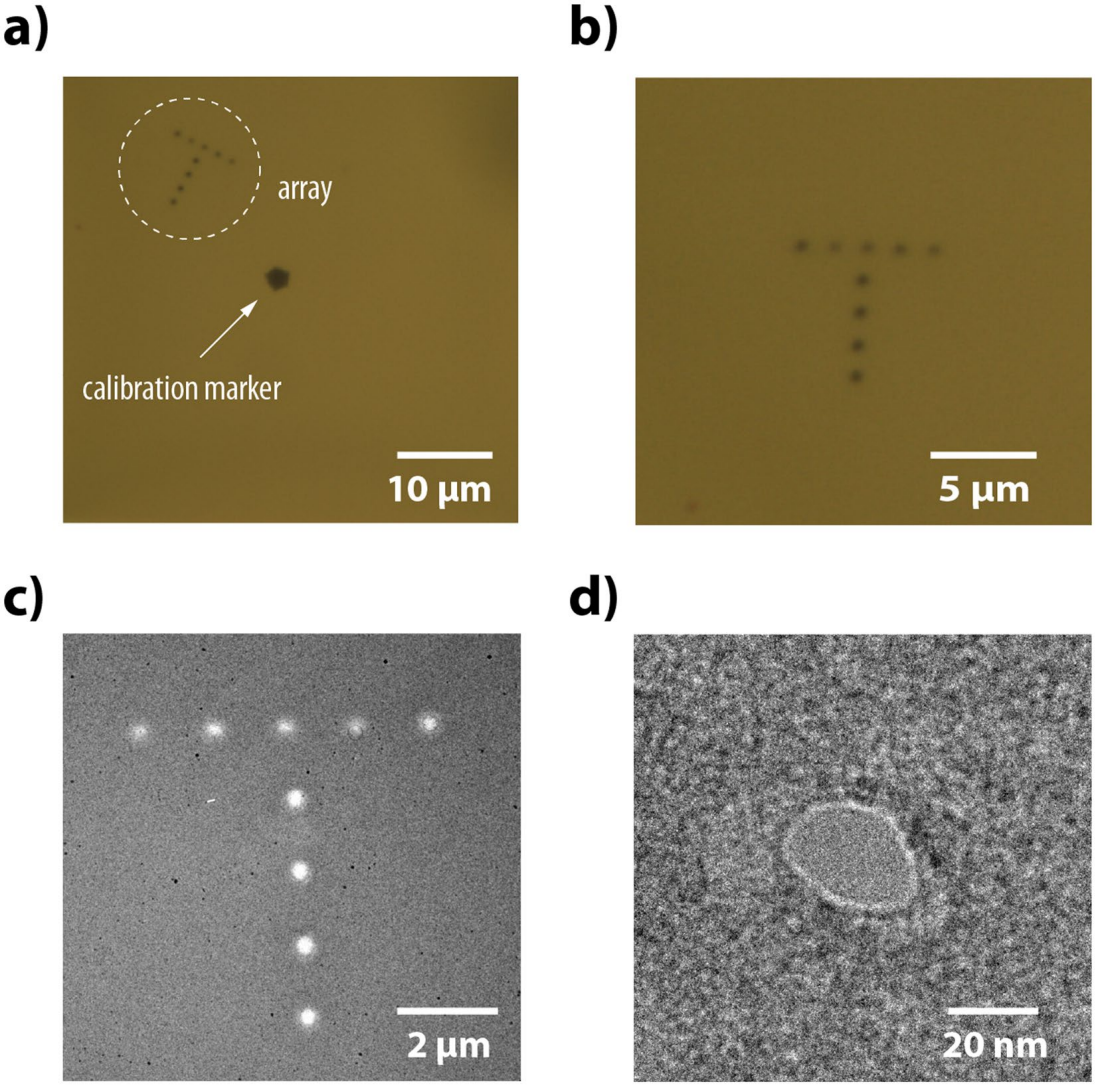
Localized laser-etching of freestanding SiN_x_. (**a**) Laser-etched T-shape array of 9 thin regions spaced 1500 ± 50 nm center-to-center. The top and vertical bars were etched with a laser intensity of ~30 mW and 45 mW, respectively, for 4 minutes each. Next to the T is a lithography-fabricated thin region (20 ± 2 nm) for comparison. (**b**) Zoom in of just the T. (**c**) TEM image of the T, showing a difference in brightness for the top and vertical bars, corresponding with a difference in thickness. (**d**) Nanopore formed in one of the thin regions.

This study presents a purely optical solid-state nanopore fabrication technique with unparalleled *in situ* control over the nanopore position. Local SiN_X_ thinning and subsequent pore formation are performed at any arbitrary point along the membrane by simply positioning the membrane at the tightly focused laser spot. To illustrate this, we constructed an evenly spaced, nanoscale-accurate (1500 ± 50 nm center-to-center) T-shape of 9 thin regions in a SiN_x_ membrane in just 36 minutes (Fig. 6a–c). The T was made next to a lithography-fabricated thin region (20 ± 2 nm) used for thickness calibration and to further prove that the produced contrast is due to thinning (Fig. 6a). The etch time of ~4 minutes per spot was more than enough to produce the visible contrast; under TEM inspection, we found that a ~20 nm nanopore had formed in one of the spots (Fig. 6d). The etch rate is also highly controllable. To show this, we varied the laser intensity in constructing the T-shape: ~30 mW and 45 mW for the horizontal and vertical bars, respectively. The difference in thickness is particularly noticeable in the TEM image, although the spots are not quite identical (Fig. 6c). Improvements to the optical setup (reducing stage drift, shrinking the laser focus, *etc*.) could enable finer etching for stricter applications such as electrode-embedded nanopore transistors^49,50^.

While nanopores can be formed optically in as little as one minute in 45 nm thick SiN_x_, we predict that the etch process can be further accelerated by optimization of the laser intensity, wavelength, and solution properties (salt, pH, *etc*.). As we study the underlying etch mechanism, we may discover ways to apply it to other common nanopore substrates as well, such as WS_2_^51^. Given that the technique is rapid and highly automatable—it can be monitored by the PL intensity and ionic current—we anticipate that it can be used to construct vast nanopore arrays for massively parallel optical sensing^4,10,15^. Notably, as nanopore fabrication proceeds so quickly, it would not be necessary to compromise on the thickness of the supporting membrane, as might be necessary using thickness-limited strategies such as CBD^23^. Furthermore, as a consequence of the inverted-Gaussian etch profile, these nanopores benefit from significantly improved spatial resolution and reduced background PL.

## Methods

### Chip fabrication

Nanopore chips were fabricated from a 4 in. silicon wafer coated with 500 nm SiO_2_ and 50 nm low-stress amorphous SiN_x_. To create freestanding membranes, a hard mask was RIE-etched into the SiN_x_ followed by HF etching to remove the SiO_2_, and then through-etching of Si with KOH. The free-standing membranes were 40–45 nm thick.

### Chip assembly

Chips were first cleaned by piranha (3:1 H_2_SO_4_:H_2_O_2_). They were then glued onto a custom-made Teflon insert, immersed in buffer (1 M KCl, 40 mM Tris-HCl, 1 mM EDTA, pH 7.5), and placed in a Teflon cell with a quartz cover-slide bottom. The cell was mounted onto a 3D nanopositioner located above the microscope objective. The setup was shielded by a grounded copper box and placed on a vibration-isolating optical table.

### Optical setup

A previously described custom-built confocal microscope^11^ was modified for this study: Three collimated laser lines are focused onto a diffraction-limited spot at the membrane surface. The emitted light is collected by the same objective (NA = 1.15), focused onto a spatial pinhole to reject out-of-focus light, passed through an ND-filter and directed onto two spectrally separated APDs for two-color imaging.

 The photoluminescence intensity was attenuated by 3 orders in magnitude, to protect the APDs, by placing an ND3 filter in the emission pathway during etching and before the excitation pathway during profiling. The photoluminescence count is a summation of the red (>650 nm) and green (550–650 nm) channels.

Ionic current was measured by *cis/trans* –immersed Ag/AgCl electrodes connected to a high-bandwidth amplifier (Axon 200B) sampled at 125 kHz (DAQ NI-6211) and filtered at 10 kHz. Photon counting was sampled at 500 kHz (DAQ NI-6602). The two cards were triggered simultaneously via a hardware connection and were fully controlled by custom LabVIEW software.

### TEM imaging

High-resolution images were acquired with an FEI Titan Themis Cs-Correct HR-S/TEM. The relative thickness map (RTM) was automatically generated using the Gatan Digital Micrograph® EFTEM technique by first acquiring an unfiltered and a zero-loss image from the same region under identical conditions. The RTM was then computed using the Poisson statistics of inelastic scattering: *t/λ* = *−ln(I*_*O*_/I_t_), where I_O_ is the zero-loss intensity and I_t_ is the total intensity. To obtain the true thickness, *t/λ* is multiplied by the mean free path (110 nm) in silicon nitride (Si:N 3:4).

### Ellipsometry measurements

Performed with model FS-1 multi-Wavelength Ellipsometer (Film Sense).

### Calcium indicator experiments

The setup and protocol exactly follows a previous study^26^, with a Fluo-4 and CaCl_2_ concentration of 500 nM and 500 mM, respectively.

### DNA Translocation experiments

Nanopores were allowed to equilibrate at a low probing voltage (0.1 to 0.3 V) in buffer (1 M KCl, 40 mM Tris-HCl, 1 mM EDTA, pH 7.5) for at least 10 minutes to obtain a stable open pore current prior to adding homemade 5054 bp dsDNA. Events were monitored using an Axon 200B filtered at 100 kHz and custom LabVIEW software.

### Protein Translocation Experiments

In some cases, nanopores were kept dry in air for up to 10 days prior to performing the experiment. These nanopores were cleaned by Dynasolve 185 to remove PDMS and then made hydrophilic by piranha (3:1 H_2_SO_4_:H_2_O_2_). Nanopores were allowed to equilibrate at a low probing voltage (0.1 to 0.3 V) in buffer (1 M KCl, 40 mM Tris-HCl, 1 mM EDTA, pH 7.0) for at least 10 minutes to obtain a stable open pore current prior to adding 0.007 μg/µl di-ubiquitin. Events were monitored using an Axon 200B filtered at 100 kHz and custom LabVIEW software.

## Electronic supplementary material

Supporting Information
