# Supplementary material for: Optically-Monitored Nanopore Fabrication Using a Focused Laser Beam
Source: Sci Rep. 2018 Jun 27;8:9765. doi: 10.1038/s41598-018-28136-z (PMC6021433; doi:10.1038/s41598-018-28136-z)
Supplement: Supplementary file 1 — Supporting Information [file 41598_2018_28136_MOESM1_ESM.pdf]

Optically-Monitored Nanopore Fabrication Using a Focused Laser Beam

Tal Gilboa[1], Adam Zrehen[1], Arik Girsault[1] and Amit Meller[1,*]

1) Department of Biomedical Engineering

The Technion – Israel Institute of Technology

Haifa, Israel 32000

*Corresponding author. E-mail: ameller@technion.ac.il

Supporting Information

Table of content:

1. TEM images of laser-etched regions
2. Laser-etching wavelength dependency
3. Additional nanopore fabrication experiments
4. DNA and protein translocation experiments

1. **TEM images of laser-etched regions**

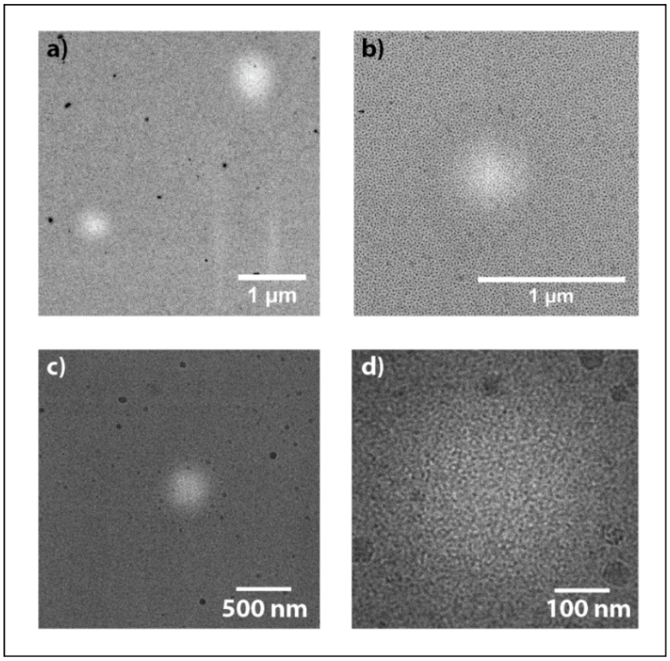

Figure S1. TEM images of laser-etched thin regions. (a) TEM image at 7500x of two thin regions corresponding to 2 different laser exposure durations: 1 and 3 minutes (left to right). (b) TEM image at 21000x of one thin region after 1 minute of laser exposure. (c) TEM image at 16500X of a thin region after 1 minute of laser exposure, and a zoom-in at 75000x (d).

2. Laser-induced etching kinetics, wavelength dependency.

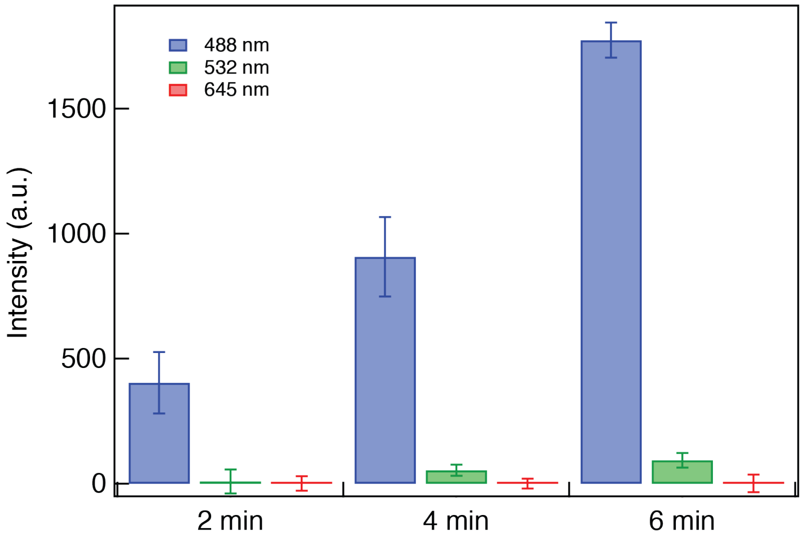

Figure S2. Laser-etching wavelength dependency. Free-standing 40-45 nm thick SiN_x were subjected to ~45 milliwatt 488 (blue), 532 (green), and 645 (red) laser intensities for 2, 4 and 6 minutes. Consequently, the membranes were imaged using a light microscope in transmission mode. The grayscale intensity values are shown as arbitrary greyscale units, obtained by averaging the pixel values at the center of the etched regions. Experiments were performed in triplicates. A higher pixel intensity corresponds to greater light transmittance and thus a thinner membrane region.

3. Additional nanopore fabrication experiments

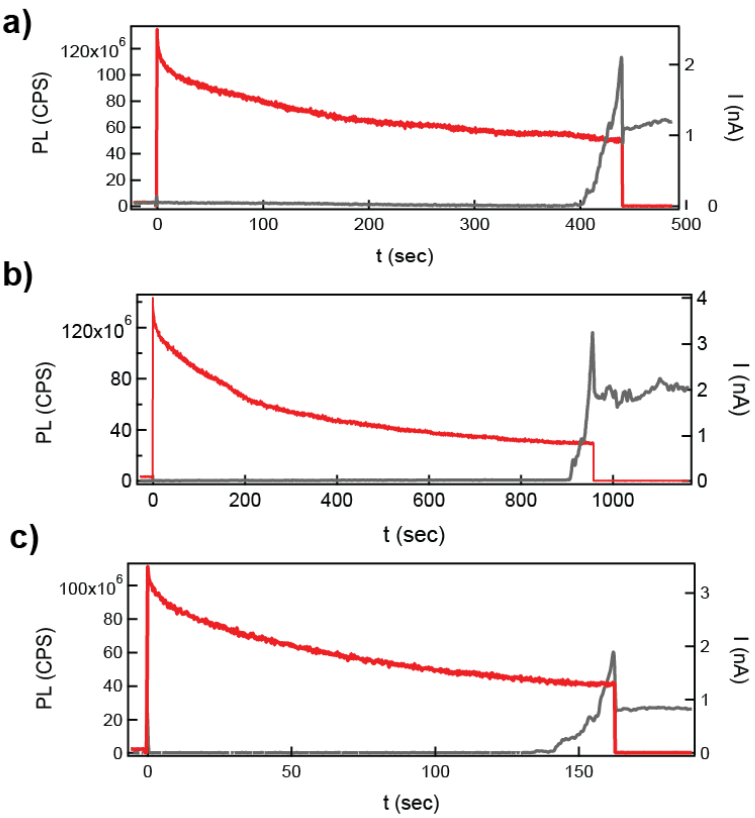

Figure S3. Nanopore fabrication by a focused laser. Three examples of measured photoluminescence (red curve) and ionic current (grey curve) during laser-exposure. A nanopore was formed after (**a**) 400 s (**b**) 920 s and (**c**) 140 s.

Pore	OPC (±0.1nA)	Pore	OPC (±0.1nA)	Pore	OPC (±0.1nA)
1	0.5	11	1.5	21	2.4
2	0.5	12	1.7	22	3.1
3	0.7	13	1.9	23	3.6
4	0.8	14	2.0	24	3.9
5	0.9	15	2.1	25	4.3
6	1.1	16	2.1	26	4.4
7	1.1	17	2.1	27	5.4
8	1.2	18	2.2	28	5.8
9	1.3	19	2.2	29	6.1
10	1.3	20	2.2	30	8.1

Table S1. Table of nanopores fabricated by laser-etching. The table is ordered according to the open pore current (OPC) from smallest to largest. In order to show that a wide distribution of pore sizes is possible, the laser was kept on following pore creation to expand the pore. The open pore current (OPC) was recorded 1-2 minutes after the laser was turned off.

4. DNA and protein translocations

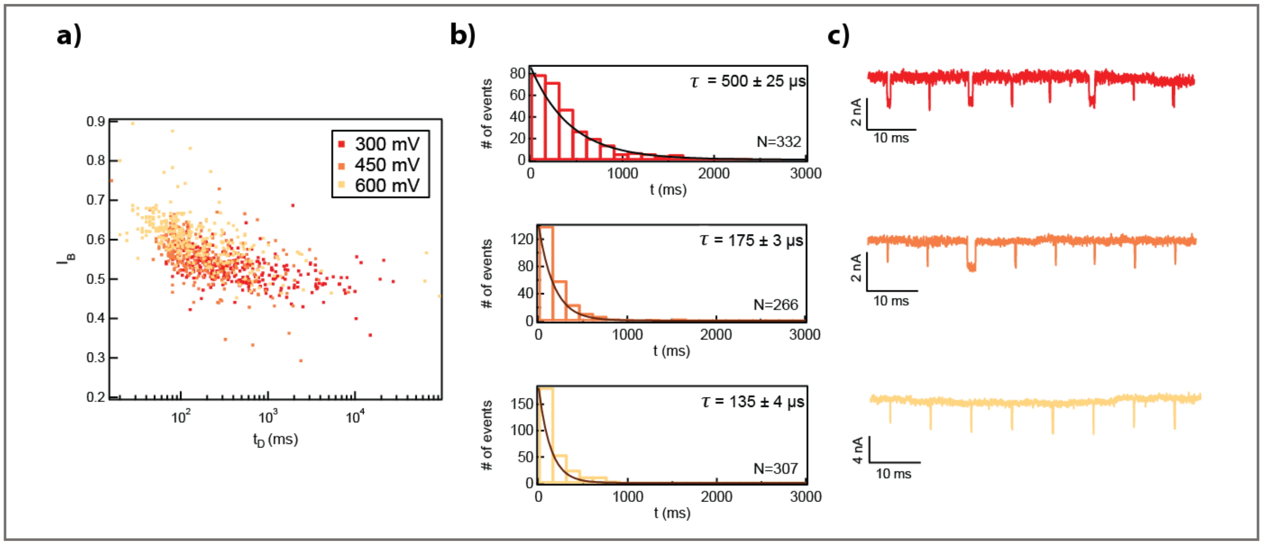

Figure S4. Translocations of 5054 bp DNA at different applied voltages: 300 mV (red), 450 mV (orange) and 600 mV (yellow). (**a**) Scatter plot of normalized translocation event blockage versus dwell time. (**b**) Translocation dwell-time histograms showing voltage-dependent time constants of 500 ± 25, 175 ± 3, and 135 ± 4 μs from lowest to highest voltage. (**c**) Sample concatenated translocation events at each voltage.

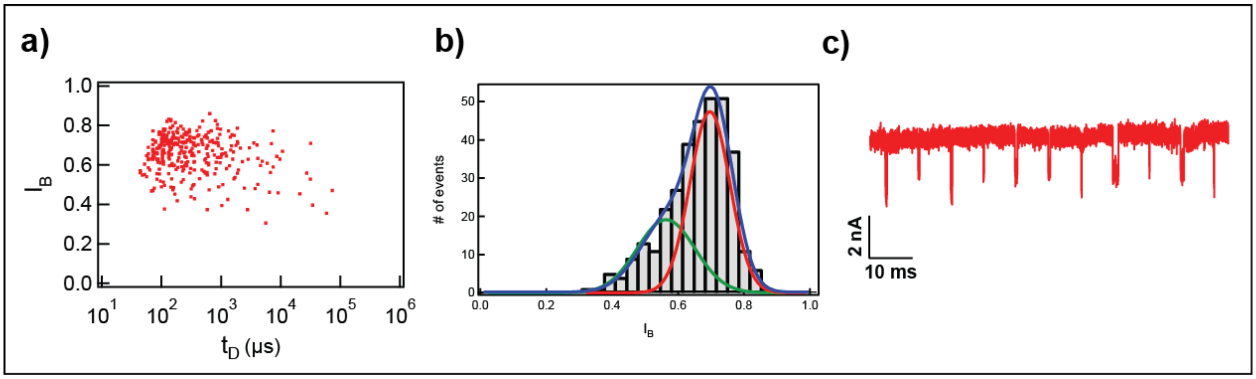

Figure S5. Translocations of 5054 bp at 300 mV. (**a**) Scatter plot of normalized translocation event blockage versus dwell time. (**b**) Normalized translocation blockage histogram fitted by two Gaussians: $\langle I_B \rangle$=0.57 (green curve) and $\langle I_B \rangle$=0.7 (red curve) (**c**) Sample concatenated translocation events.

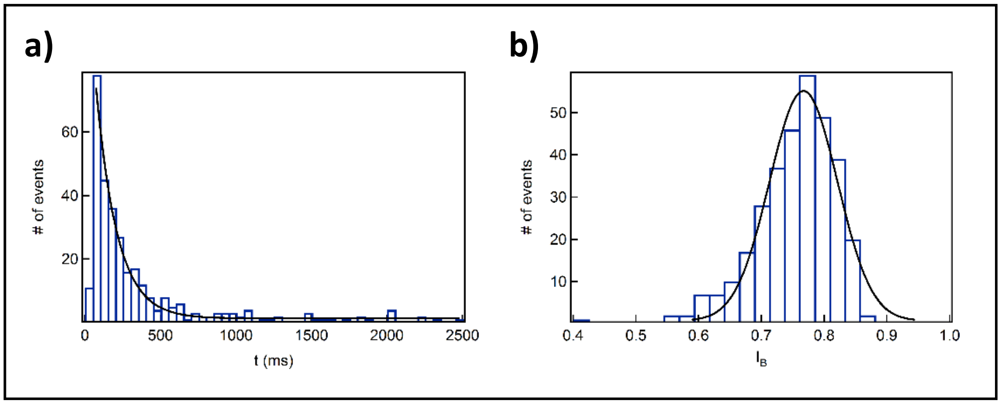

Figure S6. Translocations of di-ubiquitin (K63-linked Di-Ub) at 300 mV. (**a**) Translocation dwell-time histogram showing a decay time constant of 139 ± 10 μs. (**b**) Normalized translocation blockage histogram fitted by a Gaussian: $\langle I_B \rangle = 0.77$. N=326 events.
